# Presbyopia correction after previous Intracor treatment: Combined implantation of a small-aperture and a non-diffractive extended-depth-of-focus lens

**DOI:** 10.1016/j.ajoc.2022.101398

**Published:** 2022-02-04

**Authors:** Isabella D. Baur, Gerd U. Auffarth, Grzegorz Łabuz, Christian S. Mayer, Ramin Khoramnia

**Affiliations:** Department of Ophthalmology, University of Heidelberg, Heidelberg, Germany

**Keywords:** Cataract surgery, Refractive surgery, Extended depth of focus IOL, Small-aperture IOL, Spectacle independence

## Abstract

**Purpose:**

We present the case of implantation of two different Extended depth of focus intraocular lenses (EDoF IOLs) in a patient with a history of unilateral intrastromal femtosecond laser treatment for presbyopia correction (Intracor).

**Observations:**

The patient reported decreasing visual acuity at near distance and increasing spectacle dependence. Ten years earlier, he had Intracor treatment for presbyopia correction in his left eye. Corrected distance visual acuity (CDVA) was 0.08 logMAR for the right eye and 0.16 logMAR for the left eye. Apart from dysfunctional lens syndrome, the examination results were unremarkable. Phacoemulsification and subsequent IOL implantation was performed in both eyes. The left eye was implanted with an IC-8 (AcuFocus, Irvine, CA, USA), whereas the fellow eye was implanted with an AcrySof IQ Vivity IOL (Alcon, Fort Worth, TX, USA). Postoperatively, CDVA improved to 0.02 and 0.04 logMAR for the right and left eye. Uncorrected intermediate visual acuity (UIVA) was 0.24 logMAR for the right eye and −0.04 logMAR for the left eye, binocular UIVA was −0.04 logMAR. The patient reported a low level of photic phenomena and spectacle independence for far and intermediate distance.

**Conclusions and importance:**

Combined implantation of a non-diffractive and a small-aperture EDoF lens after previous unilateral Intracor treatment could successfully improve visual acuity at far and intermediate distance.

## 1 Introduction

Intracor intrastromal femtosecond laser treatment for presbyopia correction creates a pattern of typically five concentric rings in the corneal stroma, producing a steepening of the central cornea which improves near visual acuity. In most patients, only the non-dominant eye is treated.^1^, ^2^, ^3^ Intracor patients may continue to desire spectacle independence when presbyopia increases, or they develop age-related cataracts. Not all patients who underwent Intracor treatment achieved a near visual acuity that allowed them to be independent from reading glasses.^1^, ^2^, ^3^ Although the effect of the Intracor treatment is still present after cataract surgery or refractive lens exchange, it may not be sufficient to allow for spectacle independence in a pseudophakic patient. The question then arises, which of the numerous types of intraocular lenses (IOLs) is suitable when performing refractive lens exchange or cataract surgery? With this case report we show an exemplary solution for this problem.

## 2 Case report

A 66-year-old male patient presented to our clinic for subjective decreasing near visual acuity. The patient reported that he needed reading glasses for near distance. Ten years earlier, he had Intracor intrastromal femtosecond laser treatment for presbyopia correction on his left eye.

The results of the preoperative visual acuity testing at different distances are summarized in Table 1.

**Table 1:** Results of preoperative visual acuity testing in logMAR. OD: right eye, OS: left eye, OU: both eyes, UDVA: uncorrected distance visual acuity, CDVA: corrected distance visual acuity, UNVA: uncorrected near visual acuity, DCNVA: distance corrected near visual acuity.

	Subjective refraction	UDVA	CDVA	UNVA	DCNVA
OD	+1.75/-0.50/80°	0.70	0.08	0.80	**0.50**
OS	+1.75/−/−	0.30	0.16	0.56	**0.30**
OU		0.20	0.04	0.62	**0.32**

**Fig. 1:**
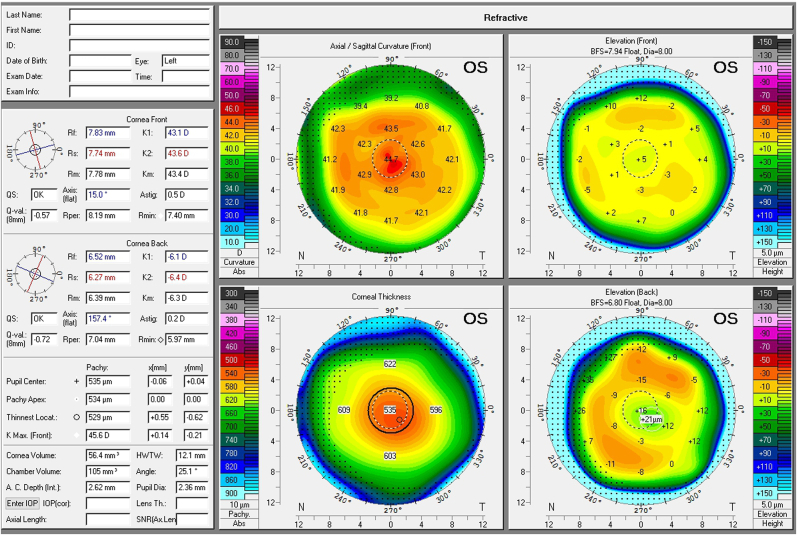
Corneal tomography of the left eye acquired with the Oculus Pentacam (OCULUS Optikgerate GmbH, Wetzlar, Germany). The image shows the central corneal steepening that is typically found after Intracor treatment.

Slit lamp examination did not reveal visually relevant cataract in either eye, therefore the finding was classified as dysfunctional lens syndrome. Five concentric intrastromal ring cuts were observed in the cornea of the left eye. Fig. 1 shows the corneal tomography of the patient's left eye acquired with the Pentacam device (OCULUS Optikgeräte GmbH, Wetzlar, Germany). The central steepening of the cornea is the result of the Intracor treatment. Funduscopy showed normal findings for both eyes.

The patient was informed in detail about the possible benefits and complications that would arise from different posterior chamber intraocular lenses, including trifocal, EDoF and monofocal IOLs. The possible complications discussed included intraoperative posterior capsular rupture necessitating implantation of a monofocal IOL. The patient was informed about potential consequences of the previous corneal surgery on the left eye, the poorer reliability of the optical biometry and an increased risk of deviation from the target refraction. Given the patient's wish for resumption of spectacle independence, we proposed implanting a small-aperture IOL in the (Intracor) left eye and a non-diffractive EDoF IOL in the right eye. After careful consideration, the patient opted for this procedure.

Optical biometry was performed with the IOL Master 700 (Carl Zeiss Meditec, Jena, Germany). For the IC-8, the Barrett Universal II formula was used without a correction factor. Following the manufacturer's recommendation to target a myopic refraction of approximately −0.75 diopters,^4^, ^5^, ^6^, ^7^, ^8^ we chose a target refraction of −0.8 diopters for the left eye. For the right eye, we used the Haigis formula for IOL power calculation of the AcrySof IQ Vivity IOL. The target refraction was the myopic refraction closest to zero.

The patient had a femtosecond laser-assisted phacoemulsification procedure for each eye. The LenSx Laser, (Alcon, Fort Worth, TX, USA) was used for capsulorhexis and lens fragmentation followed by nucleus phacoemulsification and aspiration of the cortex using the Centurion Vision System (Alcon, Fort Worth, TX, USA). In the left eye, an IC-8 (AcuFocus, Irvine, CA, USA) was implanted with an IOL power of +22.5 diopters. Two weeks after that surgery, an AcrySof IQ Vivity IOL (Alcon, Fort Worth, TX, USA) with a calculated IOL power of +20.5 diopters was implanted in the right eye.

To optimize the refractive outcome and minimize the corneal astigmatism, we used the Verion digital marking system (Alcon, Fort Worth, TX, USA) to place the main incisions at 90° for the right eye and 71° for the left eye. The intraoperative and postoperative course was uneventful.

**Fig. 2:**
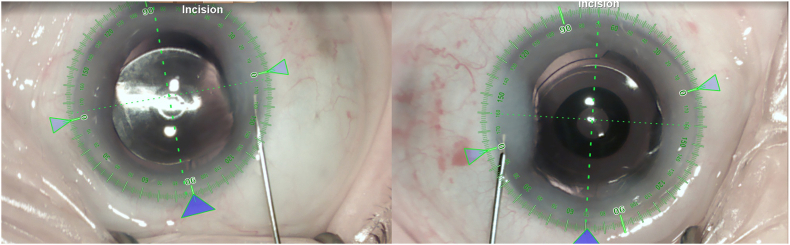
Intraoperative image with displayed digital marking system of the right eye (left part of the image) and the left eye (right part of the image).

**Fig. 3:**
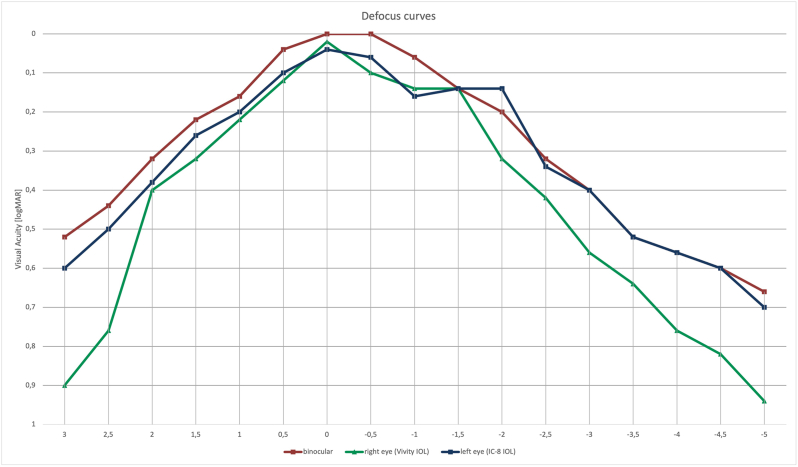
Monocular and binocular defocus curves. Defocus curve testing was performed with distance correction. For binocular testing, visual acuity was 0.2 logMAR or better from +1.25 to −2.0 diopters.

Fig. 2 shows intraoperative images of both eyes. Table 2 shows the results of visual acuity testing at different distances at the 3-month follow-up visit. Fig. 3 shows the defocus curves for monocular and binocular testing with distance correction. For binocular testing, visual acuity was 0.2 logMAR or better from +1.25 to −2.0 diopters.

**Table 2:** Results of visual acuity testing at 3 months postoperatively in logMAR. OD: right eye, OS: left eye, OU: both eyes, UDVA: uncorrected distance visual acuity, CDVA: corrected distance visual acuity, UNVA: uncorrected near visual acuity, DCNVA: distance corrected near visual acuity.

	Subjective refraction	UDVA	CDVA	UNVA	DCNVA	UIVA	DCIVA
OD	+0.25/-0.25/100°	0.08	0.02	0.54	0.60	0.24	0.14
OS	−0.50/-0.25/70°	0.16	0.04	0.32	0.40	−0.04	0.20
OU		0.02	−0.02	0.24	0.34	−0.04	0.12

**Fig. 4:**
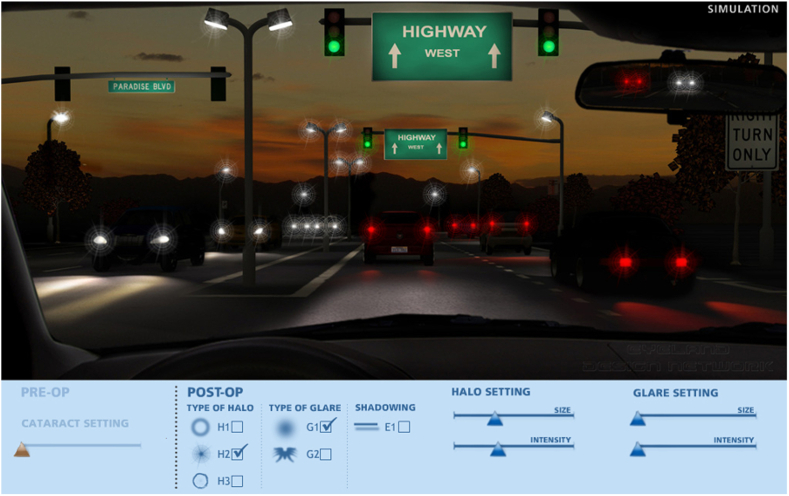
Result of the Halo & Glare simulation (Eyeland-Design Network GmbH, Vreden, Germany). The patient reported a starburst type halo (size 34 and intensity 37 on a scale from 0 to 100). The patient did not perceive glare.

To evaluate photic phenomena, we used a Halo & Glare simulator (Eyeland-Design Network GmbH, Vreden, Germany) which allows the patient to choose between three types of halos (classic halo, starburst, and irregular halo). The patient was asked to adjust the size (from 0 to 100) and intensity (from 0 to 100) according to his own perception. For glare simulation, the simulator allows two different shapes (classic glare and asymmetric glare). The patient could adjust intensity and size of the glare parameter in the same way as for the halo parameter. At the three-month examination, the patient did not experience glare. For the halo parameter, he reported the starburst type with a size of 34 and intensity of 37. The result of the simulation is shown in Fig. 4.

The patient reported he was very satisfied with the result and he reported spectacle independence for far and intermediate distance. He only occasionally used reading glasses (+1.25 diopters) for near distance.

## 3 Discussion

We observed good functional results in our patient for far, intermediate and near distance. The patient reported a low level of photic phenomena and tolerated well the implantation combining a non-diffractive EDoF and a small-aperture IOL. Due to a high level of spectacle independence, the patient was satisfied with the result.

Previous corneal surgery such as LASIK or photorefractive keratectomy for myopia in cataract patients may lead to an underestimation of the required IOL power^9^^,^^10^ Therefore, the formula for IOL power calculation has to be adjusted in these cases.^11^^,^^12^ Although even multifocal IOLs can be implanted after previous refractive surgery on the cornea,^13^ the inaccuracy of IOL power calculation can cause dissatisfaction in patients already expecting similar or better results to their earlier experience from refractive surgery. However, there is evidence that an adjustment of the IOL power calculation is not necessary in cases with previous Intracor treatment.^14^^,^^15^ Rabsilber et al. compared the results of IOL power calculation after Intracor treatment to the results derived from clinical history method, an approach that takes into account the preoperative biometry data and changes in the manifest refraction, and they did not find a statistically significant difference.^14^ A published case of a patient who underwent cataract surgery with implantation of a monofocal IOL using unaltered, standard IOL power calculation formulae also confirmed this result.^15^

The highest level of spectacle independence can be achieved with trifocal IOLs.^16^^,^^17^ They provide better optical quality at intermediate distance than bifocal IOLs and they are superior to monofocal IOLs at near and intermediate distance.^18^^,^^19^ Reading acuity at near and intermediate distance can be successfully restored after trifocal IOL implantation.^20^ However, trifocal diffractive IOLs are known to reduce mesopic contrast sensitivity and they can have increased sensitivity to glare. This arises from the optical principle of diffractive IOLs: the light is distributed between several foci and creates a superimposition of images on the retina. The scattering of light creates glare and the distribution of light leads to a reduced contrast sensitivity.^21^^,^^22^ The same problems have been reported for the Intracor treatment.^23^ Therefore, we concluded that a combination of both principles, implanting a trifocal lens in a post-Intracor eye, would give unfavorable results.

We discussed the possibility of combining a trifocal IOL in the untreated eye with a monofocal or EDoF IOL in the fellow eye with our patient. Although the data on monocular implantation of a diffractive trifocal IOL is limited, previous reports suggest that optimal results can only be expected after binocular implantation of trifocal IOLs. A study comparing unilateral to bilateral implantation of a diffractive multifocal IOL, the AcrySof SN60D3 ReSTOR (Alcon, Fort Worth, TX, USA) found significantly better functional results and patient satisfaction for the bilateral implantation.^24^ For bilateral multifocal IOL implantation, better binocular functions^25^ and a higher spectacle independence^26^ have been reported compared to unilateral implantation. Therefore, we decided to opt for a different approach.

We chose to combine two different models of extended depth of focus (EDoF) IOLs. The EDoF IOLs are a heterogenous category, they extend the visual range from far to intermediate distance, but often using different optical principles. Thus, there are diffractive, refractive and pinhole lens designs. The performance and induced side effects varies between different EDoF IOL models. The American Academy of Ophthalmology has attempted to standardize the classification as ‘EDoF IOL’.^27^ The criteria proposed include a CDVA non-inferior and DCIVA superior to a monofocal control IOL as well as a depth-of-focus that is at least 0.5 diopters greater than the control IOL.^27^

A meta-analysis by Liu et al. compared three different trifocal models (PanOptix [Alcon, Fort Worth, TX, USA], FineVison [PhysIOL, Liège, Belgium] and Lisa tri 839MP [Zeiss, Oberkochen, Germany]) and two monofocal IOL models (Tecnis ZCB00 [Johnson & Johnson, New Brunswick, New Jersey, USA] and AcrySof SN60WF [Alcon, Fort Worth, TX, USA]) to an EDoF IOL model (Tecnis Symfony ZXR00 [Johnson & Johnson, New Brunswick, New Jersey, USA]). The study found that the Symfony was superior to the monofocal IOLs at near and intermediate distance, but the trifocal IOLs provided even better results at near distance. Contrast sensitivity testing, however, was best for the monofocal IOLs, followed by the EDoF IOL. The trifocal IOLs were inferior to both the monofocal group and the Symfony IOL regarding contrast sensitivity.^28^ Symfony's spectral dependence was observed to affect visual acuity and contrast sensitivity.^29^^,^^30^

Certain EDoF IOLs such as the Mini Well (SIFI, Catania, Italy) induce less photic phenomena than trifocal diffractive IOLs.^31^, ^32^, ^33^ For the AcrySof IQ Vivity, the FDA safety and effectiveness data indicate, that there was no difference in dysphotopsia between the EDoF and the monofocal control lens.^34^ For the Symfony IOL, however, several studies found no difference between the EDoF and trifocal IOLs regarding the level of dysphotopsia induced.^28^^,^^35^, ^36^, ^37^

The implantation of the small-aperture IOL we used, the AcuFocus IC-8, and a monofocal or bifocal IOL in the fellow eye has been performed successfully in several studies.^7^^,^^8^^,^^38^^,^^39^ The IC-8 features a black mask with a diameter of 3.23 mm and a central aperture of 1.36 mm, that enhances the depth of focus using the pinhole effect. Studies reported good results for far and intermediate distance and a low level of photic phenomena.^7^^,^^38^^,^^39^ It has been shown that IC-8 has a particularly high tolerance to astigmatism and corneal irregularities, including those derived from previous hyperopic or myopic LASIK, keratoconus and corneal scars.^40^, ^41^, ^42^ We specifically opted for this IOL because of the irregular cornea (central steepening due to Intracor) in our patient's left eye. Although there are reports about bilateral implantation of the IC-8 IOL, contrast sensitivity and night driving might be affected in such cases. Also, although intermediate and near visual acuity was superior in patients after binocular implantation of the IC-8 IOL compared to patients after monocular implantation, Dick et al. found significantly higher overall patient satisfaction in the monocular group.^39^ Patients who underwent binocular implantation of the IC-8 perceived significantly more halos.^39^ Therefore, we opted not to implant bilateral IC-8 and we chose a different IOL in the patient's untreated second eye, a lens to further extend the visual range and increase spectacle independence. The AcrySof IQ Vivity IOL extends the patients visual range from far to intermediate distance using a non-diffractive Wavefront-Shaping Technology (X-Wave technology). The central part of the optic features two transition elements that stretch the wavefront to create a continuous focal range and shift the light from the hyperopic to the myopic direction to utilize all the light energy. We recently published cases of two young patients with unilateral implantation with the Vivity IOL, where we observed good tolerance to the unilateral implantation and good functional results for far and intermediate distances. These patients did not report disturbing photic phenomena.^43^^,^^44^

We targeted a slightly myopic refraction in the non-dominant left eye for the IC-8 IOL as described in previous reports and recommended by the manufacturer.^4^, ^5^, ^6^, ^7^, ^8^ The postoperative myopic refractive error created a mini-monovision additionally enhancing uncorrected near and intermediate visual acuity.

Corneal pathologies or irregularities resulting from previous surgeries can increase the risk of deviation from the target refraction.^12^ In such cases, since EDoF IOLs show greater tolerance to postoperative refractive errors,^45^ it may be advantageous to implant an EDoF IOL.

Although we were very satisfied with the success of the treatment, this was the first time we used these specific combinations of presbyopia correcting IOLs. Therefore, our results should be verified in a larger sample size.

## 4 Conclusion

The combined implantation of a small-aperture lens in the eye of a patient with previous unilateral intrastromal femtosecond laser treatment for presbyopia correction and, in his fellow eye, a non-diffractive EDoF lens provided good visual acuity outcomes for far and intermediate distances and functional results for near visual acuity. The level of dysphotopsia described by the patient was low and we observed a high patient satisfaction.

## Patient consent

Consent to publish the case report was not obtained. This report does not contain any personal information that could lead to the identification of the patient.

## Funding

The publication fee was covered by AcuFocus. Isabella D. Baur is funded by the Rahel Goitein-Straus- Programme of Heidelberg University, Faculty of Medicine.

## Authorship

All authors attest that they meet the current ICMJE criteria for authorship.

## Intellectual property

We confirm that we have given due consideration to the protection of intellectual property associated with this work and that there are no impediments to publication, including the timing of publication, with respect to intellectual property. In so doing we confirm that we have followed the regulations of our institutions concerning intellectual property.

## Research ethics

We further confirm that any aspect of the work covered in this manuscript that has involved human patients has been conducted with the ethical approval of all relevant bodies and that such approvals are acknowledged within the manuscript.

Written consent to publish potentially identifying information, such as details or the case and photographs, was obtained from the patient(s) or their legal guardian(s).

## Authorship

The International Committee of Medical Journal Editors (ICMJE) recommends that authorship be based on the following four criteria:

Substantial contributions to the conception or design of the work; or the acquisition, analysis, or interpretation of data for the work; AND, Drafting the work or revising it critically for important intellectual content; AND, Final approval of the version to be published; AND, Agreement to be accountable for all aspects of the work in ensuring that questions related to the accuracy or integrity of any part of the work are appropriately investigated and resolved.All listed authors meet the ICMJE criteria.  We attest that all authors contributed significantly to the creation of this manuscript, each having fulfilled criteria as established by the ICMJE. We confirm that the manuscript has been read and approved by all named authors. We confirm that the order of authors listed in the manuscript has been approved by all named authors.

## Contact with the editorial office

This author submitted this manuscript using his/her account in EVISE.

We understand that this Corresponding Author is the sole contact for the Editorial process (including EVISE and direct communications with the office). He/she is responsible for communicating with the other authors about progress, submissions of revisions and final approval of proofs.

We confirm that the email address shown below is accessible by the Corresponding Author, is the address to which Corresponding Author's EVISE account is linked, and has been configured to accept email from the editorial office of American Journal of Ophthalmology Case Reports:

## Declaration of competing interest

G. Auffarth and R. Khoramnia report grants, personal fees, and non-financial support from 10.13039/100007816Alcon, Acufocus and Johnson&Johnson. I. Baur, G. Łabuz and C. Mayer have nothing to disclose.
